# Self-Assembled Monolayer-Based Hole-Transporting Materials for Perovskite Solar Cells

**DOI:** 10.3390/nano14020175

**Published:** 2024-01-12

**Authors:** Doyeong Yeo, Juyeon Shin, Dabit Kim, Jae Yun Jaung, In Hwan Jung

**Affiliations:** Department of Organic and Nano Engineering, and Human-Tech Convergence Program, Hanyang University, 222, Wangsimni-ro, Seongdong-gu, Seoul 04763, Republic of Korea; dyy419@hanyang.ac.kr (D.Y.); sinju00@hanyang.ac.kr (J.S.); dabit98@hanyang.ac.kr (D.K.)

**Keywords:** self-assembled monolayers, hole-transporting materials, perovskite solar cells, anchoring group, surface modification

## Abstract

Ever since self-assembled monolayers (SAMs) were adopted as hole-transporting layers (HTL) for perovskite solar cells (PSCs), numerous SAMs for HTL have been synthesized and reported. SAMs offer several unique advantages including relatively simple synthesis, straightforward molecular engineering, effective surface modification using small amounts of molecules, and suitability for large-area device fabrication. In this review, we discuss recent developments of SAM-based hole-transporting materials (HTMs) for PSCs. Notably, in this article, SAM-based HTMs have been categorized by similarity of synthesis to provide general information for building a SAM structure. SAMs are composed of head, linker, and anchoring groups, and the selection of anchoring groups is key to design the synthetic procedure of SAM-based HTMs. In addition, the working mechanism of SAM-based HTMs has been visualized and explained to provide inspiration for finding new head and anchoring groups that have not yet been explored. Furthermore, both photovoltaic properties and device stabilities have been discussed and summarized, expanding reader’s understanding of the relationship between the structure and performance of SAMs-based PSCs.

## 1. Introduction

Perovskite crystals are one of the hottest materials for solution-processible electronic devices such as solar cells, photodetectors, transistors, and light-emitting diodes due to their high performance, which is comparable to that of existing silicon-based devices [1,2,3,4,5,6,7,8,9,10,11,12,13,14]. Among the various applications, perovskite solar cells (PSCs) have achieved remarkable progress, and the power conversion efficiency (PCE) of PSCs has dramatically increased over the past decade, now reaching a PCE value of 25.2%, which is comparable to that of commercialized silicon solar cells [15,16,17,18,19,20,21,22,23,24]. After the first report on PSCs in 2009 [25], there have been many attempts to improve the efficiency and long-term stability of PSCs, such as interface engineering, perovskite composition optimization and encapsulation techniques, and so on [26,27,28,29,30]. Among those attempts, modifying interfacial layers such as the hole-transporting layer (HTL) and the electron-transporting layer (ETL) has also been widely studied. These modifications enhance the charge transfer at the interface of the electrodes and directly affect the growth of perovskite crystals on the active layer [31,32,33,34]. In particular, the role of the HTL is more critical in inverted (p-i-n) PSCs than in conventional (n-i-p) PSCs because the HTL directly affects the growth of the perovskite layer in the inverted structure [35,36].

Both inorganic and organic materials have been developed as hole-transporting materials (HTMs) for inverted PSCs. In the case of inorganic HTMs, copper(I) iodide (CuI), copper(I) thiocyanate (CuSCN), copper oxides (Cu_2_O and CuO), and nickel oxide (NiO) are currently being developed, with the great advantages of low production costs, high hole mobility, and chemical stability [37]. However, there are some limitations related to adjusting energy levels and solution processability [38]. In the case of organic HTMs, they require multiple synthetic steps from commercially available organic chemicals, but it is easy to diversify the molecular structures of materials, which allows for the fine-tuning of energy levels. In addition, they have the unique advantages of being light-weight, having a less-adverse environmental impact, and solution processability [39]. There are roughly two types of organic HTMs: (1) one comprises conventional polymer or small-molecule HTMs forming their own energy levels, and (2) the other includes small-molecule HTMs making self-assembled monolayers (SAM) capable of modifying the energy levels of electrodes. The well-known polymer-type HTMs are PTAA and PEDOT:PSS, which all possess excellent electrical conductivity with proper HOMO energy levels, facilitating hole transport from the perovskite layer to the electrode [40,41,42,43]. However, those HTMs should be deposited to a thickness of several tens of nanometers for film uniformity, and their HOMO levels and hole mobilities must be well controlled for the efficient transport of charge [44,45,46]. On the contrary, SAM-based HTMs are extremely thinly coated on the transparent conductive oxide (TCO) electrodes, forming a permanent dipole moment at the interfaces, which effectively modulates the work function (WF) of the electrode [47,48,49,50]. In addition, the strong binding affinity of the anchoring groups of SAMs effectively reduces the defect sites at the TCO surfaces, minimizing the charge recombination at the interface of the HTL and perovskite layer. Moreover, hydrophobic surfaces, after SAM treatment, further improve the growth of perovskite crystals, increasing the performance of PSCs [51,52].

**Figure 1:**
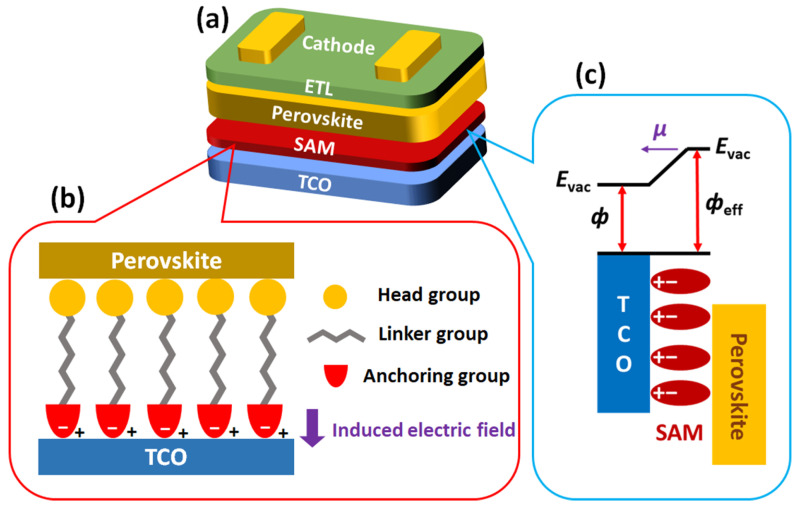
(**a**) The general device structure of inverted PSCs, (**b**) three components of HTMs forming SAM and their location in the PSCs, and (**c**) the modification of WF by SAM.

In this review, we focus on recent developments in SAM-based HTMs for PSCs, notably classifying them based on their synthetic similarity to provide general information for building SAM structures. SAMs are composed of head, linker, and anchoring groups, and the selection of anchoring groups is key to designing the synthetic procedure of SAM-based HTMs. Thus, we classify SAM-based HTMs by anchoring groups such as phosphonic acid (PA), carboxylic acid (CA), and cyanoacetic acid (CAA) that attach to a TCO electrode. The anchoring groups play a crucial role in determining the binding energy at the surface of the electrodes and in influencing the interfacial dipole moment as well as the charge transport and recombination in the devices [38,50,53,54]. The related working mechanism of SAM-based HTMs is visualized and explained (Figure 1) to provide inspiration for finding new head and anchoring groups that have not yet been explored. In addition, both photovoltaic properties and device stabilities are discussed and summarized in detail, which expand reader’s understanding of the relationship between the structure and performance of SAMs-based HTMs for PSCs. We hope that this review will provide some insight into the different types of head and anchoring groups and consequently contribute to further progress in the development of SAM-based PSCs.

## 2. SAM-Based HTMs

### 2.1. Structure and Characteristics of SAM-Based HTMs

The structure of the HTMs forming SAMs is categorized into three components, as shown in Figure 1. The first part is a head group in direct contact with the perovskite layer. Head groups are typically composed of hydrophobic conjugated molecules, such as triphenyl amine, carbazole, and phenothiazine, which improve contact with the perovskite layer compared to bare TCO [55]. The second part is an anchoring group that binds to the TCO electrodes, such as indium tin oxide (ITO) or fluorine-doped tin oxide (FTO). The interaction between the anchoring group and the TCO creates an induced electric field at the interfaces, which down-shift the WF (increase the effective WF, *ф*_eff_) of the TCO for efficient hole transport [34,56]. There are various anchoring groups, such as PA, CA, CAA, B(OH)_2_, and -SO_3_H. Among them, PA, CA, and CAA are dominantly utilized to build SAM-based HTMs for PSCs. The third part of the structure is a linker group that connects the head group and the anchoring group. Both aliphatic and aromatic groups can be used as linkers.

The synthesis of SAMs starts with the design of the core head group, which primarily employs electron-donating moieties. After that, the anchoring group is introduced into the head group with a proper space linker. The synthetic routes required to build SAM-based HTMs are mainly determined depending on the anchoring groups, and, thus, we categorized them by the anchoring groups, as follows. (1) PA anchoring group: First, the electron-donating core unit is brominated, and then it reacts with triethylphosphate to produce phosphonate. Then, a hydrolysis procedure is subsequently used to convert it to phosphonic acid. (2) CA anchoring group: CA anchoring groups can be incorporated into SAM-based HTMs in two different ways. The first way is introducing a benzoic acid group at the end of the HTMs. The head and linker are connected first, and then alkyl benzoate is reacted with by C-C coupling in the presence of a palladium catalyst. Subsequently, the resulting compound undergoes hydrolysis, followed by an acid treatment, to ultimately convert the ester group into a CA group. The second way consists of introducing an aliphatic CA to the HTMs. The aliphatic ester is alkylated to the electron-rich head group, usually including amine derivatives. The ester group is transformed into a CA group through a saponification reaction. (3) CAA anchoring group: Initially, an aromatic aldehyde is linked to the electron-rich head group via palladium-catalyzed C-C coupling. And then, a CAA group is easily introduced into the resulting compound through Knoevenagel condensation.

### 2.2. PA-Based SAMs

**Figure 2:**
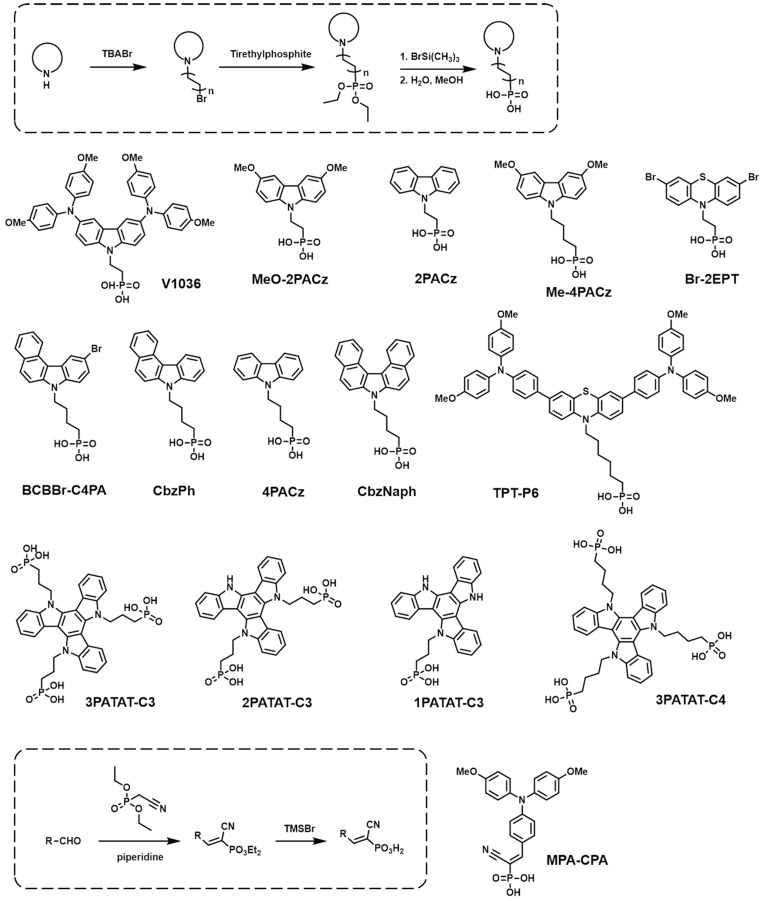
General synthetic routes and molecular structures of **PA** anchoring group-based SAMs.

The PA group has been demonstrated to create strong and stable bindings on TCO surfaces, enabling efficient WF modifications [57,58]. This group contains two hydroxy groups and one phosphonic group, allowing for three different binding modes depending on the substrate surfaces and reaction conditions [38]. Among various anchoring groups, the PA group exhibited the highest bond energy, especially with TiO_2_ surfaces [59]. These strong bonds contribute to an exceptional monolayer stability. Consequently, the PA group is considered to be one of the most powerful anchoring groups for SAMs, and numerous SAMs have been synthesized with this group in order to be used as HTMs for PSCs (Figure 2). The photovoltaic properties of PSCs are summarized in Table 1.

As the first SAM-based HTM of p-i-n PSCs, Magomedov et al. reported a **V1036** device in 2018. **V1036** is composed of a PA group as the anchoring group, ethyl as the linker, and dimethoxydiphenylamine-substituted carbazole as the head group [60]. They also mixed electrically inactive filler molecule butylphosphonic acid (C4) with **V1036** to improve the surface properties of SAMs on ITO. C4 enhances wettability and ionizability and fills pinholes, resulting in the best PCE of 17.8% at the molar ratio of **V1036:** C4 = 1:9. Moreover, the device maintained approximately 94% of its initial PCE after 180 days under N_2_, in the dark, at room temperature (RT). After that, Al-Ashouri et al. developed **MeO-2PACz** and **2PACz** using a simple carbazole head and a PA anchoring group in 2019 [61]. The **MeO-2PACz**- and **2PACz**-based PSCs exhibited enhanced performances compared to the control PTAA-based devices because SAM-based HTMs make a favorable energetic alignment with perovskite and reduce defect states at the interface, leading to a decreased non-radiative recombination in the devices. Consequently, **2PACz** showed a maximum PCE of 20.9%, a *V_OC_* of up to 1.19 V, and only a <3% PCE loss after 11 h of operation under 1 sun AM 1.5G illumination. Following this, Al-Ashouri et al. designed **Me-4PACz**, structurally similar to **MeO-2PACz** but with a butyl linker group, and the **Me-4PACz**-based PSCs showed a PCE of 20%, an FF of 84%, and a *V*oc of up to 1.15 [62]. The devices kept 95.5% of their initial PCE after 300 h in air under a relative humidity of 30 to 40%, at RT. After the remarkable achievement of SAMs based on carbazole and PA anchoring groups like **MeO-2PACz** and **2PACz**, various head groups have been explored to build SAM-based HTMs to improve the performance of PSCs. Ullah et al. synthesized **Br-2EPT** using a brominated phenothiazine moiety as the terminal unit, ethyl as the linker, and PA as the anchoring group [63]. Because of the electron-withdrawing property of phenothiazine, it lowered the highest occupied molecular orbital (HOMO) energy level, and the Br-functional group improved the hydrophobicity of the interface, increasing the crystallinity of the perovskite. When the **Br-2EPT** SAM was incorporated into inverted PSCs, it resulted in low non-radiative losses and a well-matched energy level with perovskite, leading to an impressive PCE over 22% and FF up to 80%. Moreover, the devices showed an increase in PCE from 20.3% to 21.8% after 100 h of continuous maximum power point tracking. Wang et al. reported a **BCBBr-C4PA** SAM showing a lower-lying HOMO energy level of -5.46 eV and negligible light absorption. The **BCBBr-C4PA** SAM improved the interfacial charge transfer and reduced the non-radiative recombination losses, resulting in a promising PCE of 18.63% with significantly improved operation stability (over 90% PCE retention after 250 h of continuous working) [64]. Jiang et al. synthesized two benzene ring-extended carbazole-based SAMs, **CbzPh** with asymmetric π expansion and **CbzNaph** with helical π expansion [65]. They were compared with **4PACz**, which had simple carbazole head groups. The **4PACz** and **CbzPh** did not exhibit sufficient π-π stacking interaction between the head groups, but **CbzNaph**, forming a helical configuration, led to strong π-π interactions between the head groups. As a result, employing a **CbzNaph**-based SAM as the HTM in inverted PSCs yielded an impressive PCE of 24.1% along with enhanced device stability (retaining 97% of its initial PCE after 120 h under continuous illumination). Li et al. synthesized three SAMs, **TPT-S6**, **TPT-C6**, and **TPT-P6**, featuring distinct anchoring groups of SO_3_H, CA, and PA, respectively, while all having a phenothiazine head group. Although **TPT-C6** was previously reported to be **TPA-PT-C6** by the same group in 2020, Li et al. referred to **TPA-PT-C6** as **TPT-C6** to compare the differences in anchoring groups of SAMs. The discussion on **TPA-PT-C6** (**TPT-C6**) can be found in the CA-based SAMs section (Section 2.3). It was discovered that anchoring groups with strong bonding strengths increase SAM compactness by improving the tolerance for perovskite deposition in addition to assembly rate and adsorption density. Among them, **TPT-P6**, having a PA anchoring group, showed the fastest and most robust assembly, resulting in an ordered, dense, and stable monolayer on the ITO. The TPT-P6-based PSCs achieved an increased PCE of 22% and enhanced stability, retaining 90% of their initial performance after three months. These results showed that the PA anchoring group is the most suitable for SAMs as a perovskite HTM [66].

Zhang et al. developed an amphiphilic SAM, **MPA-CPA**, with a multifunctional cyanovinyl phosphonic acid anchoring group which provides a superwetting underlayer for perovskite deposition, allowing for high-quality perovskite films with fewer defects at the interface [67]. After spin coating the **MPA-CPA** solution onto an ITO substrate, a bilayer stack consisting of a chemically anchored SAM and a non-adsorbed disordered overlay was formed. The overlayer composed of amphiphilic **MPA-CPA** exhibited superwetting properties with respect to the perovskite precursor solution and had a small contact angle (~5°) that was favorable for perovskite deposition. The presence of an overlayer was important for the superwetting properties. As a result, the MPA-CPA-based PSCs exhibited a PCE of 25.2% with a *V*_OC_ up to 1.20 eV, a fill factor (FF) of up to 84.5%, and high stability, retaining >90% of their initial performance after 2000 h. Truong et al. synthesized the **xPATAT-Cy** series, varying the number of PA functional groups in triazatruxene derivatives, where ‘x’ denotes the number of anchoring groups (1, 2, or 3), and ‘y’ indicates the number of carbon atoms in the spacer (3 or 4) [68]. When **1PATAT-C3** with one anchoring group undergoes chemical adsorption on a substrate, it typically aligns itself in such a way that its π-plane stands perpendicular (edge-on) to the surface of the metal oxide. This specific molecular orientation might lead to inefficient orbital overlap with the perovskite surface and unintentional hole transport in the lateral direction, which could result in undesirable interfacial recombination and disruption of the hole extraction process. In contrast, **2PATAT-C3** and **3PATAT-C3**, with more than one anchoring group, secure the π-conjugated backbone in a face-on orientation to the substrate, minimizing interfacial recombination and improving hole extraction. Especially, the orientation of **3PATAT-C3** is more aligned face-on to the substrate than **2PATAT-C3**. Consequently, PSCs incorporating the tripodal **3PATAT-C3** exhibited a PCE of 23.0% and showed better device performance than those incorporating **1PATAT-C3** and **2PATAT-C3**, with PCE values of 21.1% and 22.2%, respectively. In the stability test of **3PATAT-C3**-based PSCs, the devices maintained their initial PCE without any drop after 2000 h under an inert atmosphere, RT, and dark conditions. These results highlight the fact that the multipodal strategy promotes a face-on molecular orientation on the substrate, boosting PSC performance. Truong et al. also fabricated PSCs based on **3PATAT-C4,** with a longer alkyl chain. However, the PCE of the device based on **3PATAT-C4** was slightly reduced compared to those with **3PATAT-C3**, indicating that the longer insulating alkyl chains in **3PATAT-C4** hindered hole extraction.

### 2.3. CA-Based SAMs

**Figure 3:**
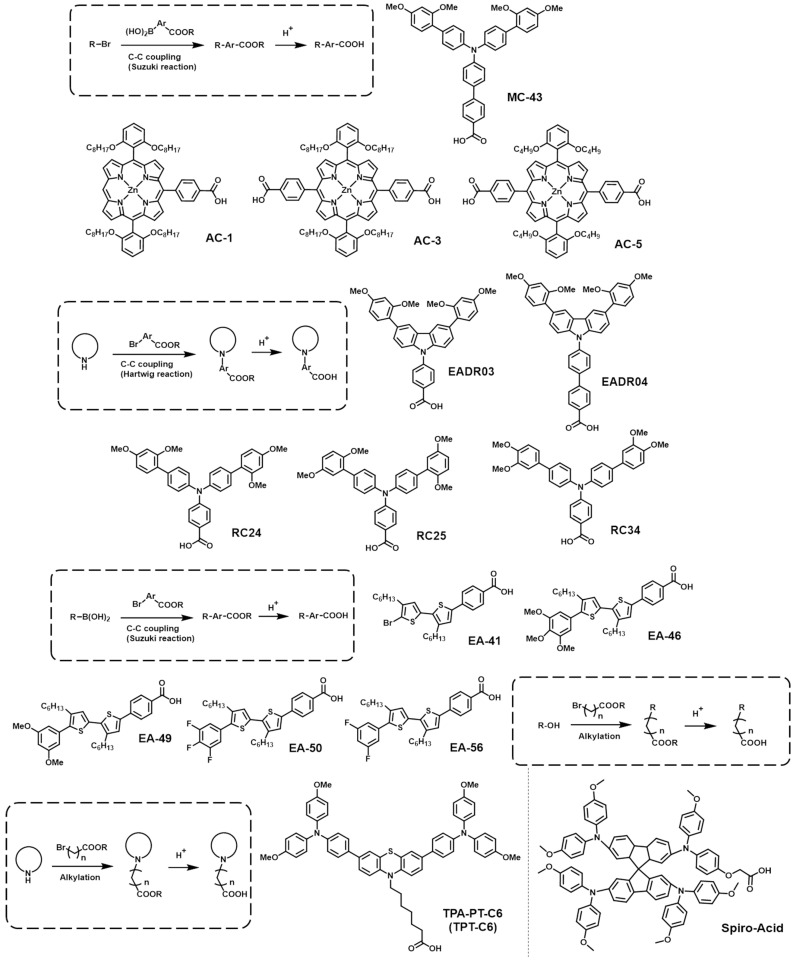
General synthetic routes and molecular structures of **CA** anchoring group-based SAMs.

The CA group is well-known for its strong binding affinity to metal oxides like ZnO and ITO, and the CA-based surface modifiers have demonstrated excellent performance in organic solar cells [69,70]. Moreover, due to the electron-withdrawing property of the CA group, it creates a strong dipole moment when combined with electron-rich moieties [71]. This characteristic could effectively modify the WF of the electrode, and, thus, SAMs with a CA anchoring group have been used as surface modifiers of the electron-transporting layer in PSCs [72]. Recently, various research results have also been reported on their use as HTMs in PSCs (Figure 3).

In 2019, Yalcin et al. synthesized a novel SAM-based HTM, **MC-43**, to compare device performance with the 4,4″-bis(diphenyl amino)-1,1′:3′,1″-terphenyl-5′-carboxylic acid (TPA) previously reported [73,74]. **MC-43** consists of a triphenyl amine head group π-extended with dimethoxy phenyl moieties, a phenyl linker group, and a CA anchoring group. These researchers fabricated devices with **MC-43**, TPA, and PEDOT:PSS, respectively, to assess the influence of polymer-based and SAMs-based HTMs on PCSs.

Notably, **MC-43** achieved the highest PCE of 17.3% among the inverted PSCs reported at that time. In addition, the device showed a high stability, maintaining 90% of its initial PCE after 20 days in dark and low-humidity conditions. These results indicate the potential of CA anchoring group-based SAMs as promising materials capable of replacing conventional polymer- and small-molecule-based HTMs. After this successful development of SAMs with a CA anchoring group, Arkan et al. reported five different SAMs by changing the electron-donating and -withdrawing effects of the head groups. The electron-donating properties of the head group increased in order of **EA-50** > **EA-56** > **EA-41** > **EA-49** > **EA-46** by adjusting the number of methoxy and fluorine moieties of the head groups [71]. As the electron-donating strength of the head group increased, the WF of the SAM-treated ITO was down-shifted, and the HOMO energy-level difference between the perovskite layer and the SAM-treated ITO layer was decreased. The reduction in their HOMO energy gap was advantageous to increasing the PCE values in the PSCs.

However, the highest PCE of 12.03% was achieved in the **EA-49**-based devices, even though the HOMO energy gap between the perovskite layer and the **EA-49**-treated ITO was not minimum. Thus, when selecting the head group of SAMs for an HTM, it is important to consider the electron-donating properties of the head groups for WF tuning as well as the interfacial interactions of the head groups with the perovskite layer. Li et al. introduced SAM-based HTMs to enhance the efficiency of large-area PSCs [75]. The newly synthesized **TPA-PT-C6** had a structure composed of a triarylamine-terminated phenothiazine head group, an alkyl linker, and a CA anchoring group. Interestingly, ammonium salt was co-assembled with **TPA-PT-C6** on the ITO substrate. This mixed-SAM system, in combination with a quaternary ammonium halide group, promoted the growth of the perovskite layer by increasing the hydrophilicity of the ITO as well as reduced the cation vacancy defects. As a result, a PCE of 17.49% was achieved for an area of 1.02 cm^2^, and a PCE of 12.67% was achieved for an aperture area of 36 cm^2^. These results demonstrate that a SAM-based HTM significantly contributes to the scaling-up of PSCs.

Aktas et al. demonstrated improved device stability of PSCs by introducing two novel SAMs as the HTM [76]. The dimethoxy-substituted phenyl ring was substituted to the carbazole moiety to increase the electron-donating effect of the head group, and the length of the linker was controlled by the number of phenyl rings. **EADR03** and **EADR04** have a phenyl and a biphenyl linker, respectively. The synthesized SAMs showed better electron-blocking properties than the control PTAA HTM, and the measured PCE value of the SAMs-based devices consistently yielded high values exceeding 21%. Notably, the SAM-based devices showed much-improved long-term stability compared to the PTAA-based devices. The PTAA-based devices reached T_80_ (time until the cell reaches 80% of its initial efficiency) in 81 h. In contrast, both SAM-based devices exhibited significantly prolonged stability, surpassing 180 h, under the same conditions. **EADR04** showed the best long-term stability with a T_80_ of over 800 h. The π-expanded linker in **EAD04** contributed greatly to its higher decomposition temperature. These results highlight the substantial role of SAMs in enhancing the stability of PSCs and suggest that stability can be further improved by controlling the linker group. After this experiment, Aktas et al. focused on the substituents in the head group of SAMs, directly facing the perovskite layer [77]. They synthesized three novel SAMs, namely **RC-24**, **RC-25**, and **RC-34**, based on TPA with two methoxy substituents positioned differently. The advantages of the methoxy group in SAMs have been demonstrated to enhance the hydrophobicity of the electrode substrates and increase the grain size of the perovskite crystals [78]. The contact angle measurements revealed that **RC-24**, with methoxy groups in the ortho and para orientation, exhibited the highest hydrophobicity, larger perovskite crystal grains, and fewer grain boundaries. Additionally, the well-ordered structure of **RC-24** improved its stability on the electrode substrates, resulting in an increased PCE of 19.8%. Hung et al. reported three Zn(II) porphyrin-based SAMs, namely **AC-1**, **AC-3**, and **AC-5** [79]. Porphyrin analogues have been utilized as HTMs in conventional (n-i-p)-type PSCs, but they have never been attempted in inverted (p-i-n) PSCs. The CA group was employed as the anchoring group of the SAMs, with **AC-1** featuring one CA group, **AC-3** featuring two CA groups, and **AC-5** with two CA groups and a shortened carbon side chain. XPS spectra, in particular, revealed that the SAMs with two anchoring groups not only reduced the WF of the ITO for a better alignment with the valence band of perovskite but also effectively reduced defects at the SAM/perovskite interface by coordinating with Pb^2+^ ions in the perovskite. The peaks of Pb^0^ in the XPS spectra decreased in **AC-3** compared to **AC-1** and were notably absent in **AC-5**. Consequently, the PSC based on **AC-5** achieved the highest PCE of 23.19%, with an FF of 84.05%. Moreover, the devices manufactured with a large area (1.96 cm^2^) exhibited a promising performance, with a PCE of 21.11% and an FF of 80.97%. With respect to storage stability, **AC-3** and **AC-5** maintained 90% and 91% of their initial PCE after one month, respectively, demonstrating better stability than the control **2PACz**, which maintained 88% of their PCE under dark conditions at RT and a relative humidity of 25% ± 5%. Li et al. synthesized modified Spiro-OMeTAD for SAMs by introducing a CA anchoring group [80]. Although Spiro-OMeTAD demonstrated an exceptional PCE of 25.8% in conventional PSCs, its application in inverted PSCs has been scarcely studied. **Spiro-Acid**, a Spiro-OMeTAD-modified SAM, is synthesized through a simple 2-step reaction without column chromatography, starting from commercially available Spiro-OMeTAD. Importantly, **Spiro-Acid** does not require any chemical doping to enhance its conductivity and hole mobility. As a result, PSCs based on **Spiro-Acid** achieved a PCE of 18.15%, which is the highest value among the inverted PSCs utilizing Spiro-OMeTAD. Furthermore, the device demonstrated a remarkable stability under long-term illumination. When subjected to simulated 1 Sun AM 1.5 G illumination for extended periods, the SAM-based device remained highly stable during the 100 h period, while the device using control PTAA dropped to 82% at the 100 h timepoint. The photovoltaic properties of PSCs are summarized in Table 2.

### 2.4. CAA-Based SAMs

Although CAA includes the CA part, it was classified separately because the synthesis of CAA-based SAMs is completely different from that of CA-based SAMs. The CAA group efficiently passivates the perovskite film because the Lewis base characteristics of CAA can trigger a coordination interaction with Pb^2+^ ion defects [81]. Furthermore, the CAA group has a stronger electron-withdrawing property than the CA group because it contains two electron-accepting moieties, -COOH and -CN. Thus, the combination with CAA and electron-donating head groups like carbazole and aryl amine forms a strong D-A structure, which is effective in enhancing hole extraction and reducing charge recombination in PSCs, making it suitable for HTM design [82]. The photovoltaic properties of PSCs are summarized in Table 3.

**Figure 4:**
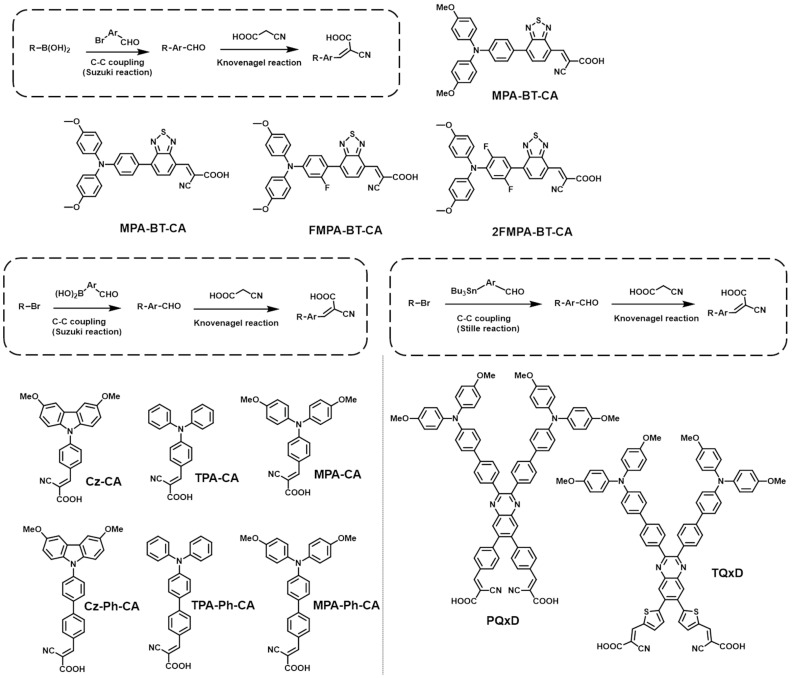
General synthetic routes and molecular structures of **CAA** anchoring group-based SAMs.

As shown in Figure 4, several SAM-based HTMs including the CAA anchoring group for PSCs are reported. Wang et al. synthesized **MPA-BT-CA** by introducing the CAA group into the HTM of PSCs for the first time, which was mainly used as dye molecules in dye-sensitized solar cells [82,83]. They employed 4-methoxy-N-(4-methoxyphenyl)-N-phenylaniline as the electron-donating moiety and benzo[c][1,2,5]-thiadiazole (BT) as the electron-accepting moiety for the head and linker groups and CAA for the anchoring group. The water contact angle of the **MPA-BT-CA**-treated ITO was 88.2°, which was 24.6° higher than that without an anchoring group. This showed that the CAA anchoring group of **MPA-BT-CA** is well-attached to the ITO substrate. Consequently, the **MPA-BT-CA**-based devices showed much a higher PCE of 21.24% than those without an anchoring group (PCE = 18.34%) and excellent long-term stability, retaining over 98% of their initial performance after 14 days under ambient air with a relative humidity of 72%. This result indicates that the CAA anchoring group plays a crucial role for SAM-based HTMs in PSCs. Following this research, Liao et al. reported two novel SAMs, namely **FMPA-BT-CA** and **2FMPA-BT-CA**, introducing fluorine atoms to **MPA-BT-CA** [84]. The incorporation of fluorine atoms resulted in reduced HOMO energy levels and larger dipole moments in both **FMPA-BT-CA** and **2FMPA-BT-CA**, facilitating the extraction of holes at the interface compared to the fluorine-free molecule **MPA-BT-CA**. Furthermore, fluorination enhanced the buried interfacial interaction between the HTL and perovskite, improving the overall device performance. As a result, the devices based on **FMPA−BT-CA** achieved remarkable performances, with an 83.3% FF and a 22.37% PCE, and better long-term stability compared to those based on **MPA-BT-CA**, maintaining over 74% of their original PCE after 120 h without encapsulation in inverted PSCs. To improve the stability of SAMs with aliphatic linker groups, Zhang et al. developed **Cz-CA** by introducing a conjugated linker between the dimethoxy carbazole head and the CAA anchoring group [85]. The synthesized **Cz-CA** demonstrated better electrochemical stability and photostability compared to **MeO-2PACz,** which had an ethyl linker. Six different SAMs were also synthesized by adjusting the length of the conjugated linker on the dimethoxy-substituted carbazole, triphenylamine, and dimethoxy-substituted triphenylamine heads. Among them, **MPA-Ph-CA**, featuring an π-extended biphenyl linker and a dimethoxy-substituted triphenylamine head, achieved the highest PCE value of 22.53% and showed excellent stability, retaining over 95% of its initial performance under continuous illumination for 800 h. Afraj et al. reported novel D-A-type quinoxaline-based SAMs, **PQx**, **TQx**, **PQxD**, and **TQxD**, for tin-based PSCs [86]. **PQxD** and **TQxD** featured the CAA anchoring group, while **POx** and **TQx** had a dicyano anchoring group. The electron-withdrawing property of the quinoxaline moiety stabilized the HOMO energy level of the SAMs, which created a better energy level alignment with the valence band of the perovskite layer. In addition, their high-lying LUMO energy levels could prevent electron transport from the perovskite layer to the anode. Moreover, the two anchoring groups on the SAMs created better contact and alignment on the ITO surface. Among the four SAMs, **TQxD**, which featured a thiophene linker and two CAA anchoring groups, exhibited the highest PCE value of 8.3% in tin-based PSCs. Notably, the SAM-based PSCs, excluding PQx, demonstrated excellent stability by maintaining 90% of their initial PCE for 1600 h.

**Table 3:** Photovoltaic properties of PSCs using **CAA**-based SAMs.

HTM	Device Structure	V_OC_ [V]	J_SC_ [mA/cm^−2^]	FF [%]	PCE [%]	Device Stability ^a^	Refs.
**MPA-BT-CA**	ITO/MPA-BT-CA/(FA_0.17_MA_0.94_PbI_3.11_)_0.95_(PbCl_2_)_0.05_/C_60_/BCP/Ag	1.13	22.25	84.8	21.24	98%, 14 d, RT ^e,g^	[82]
**MPA-BT-CA**	ITO/MPA-BT-CA/(FA_0.17_MA_0.94_PbI_3.11_)_0.95_(PbCl_2_)_0.05_ /C_60_/BCP/Cu	1.10	22.73	82.3	20.70	67%, 120 h, RT ^d,g^	[84]
**FMPA-BT-CA**	ITO/FMPA-BT-CA/(FA_0.17_MA_0.94_PbI_3.11_)_0.95_(PbCl_2_)_0.05_/C_60_/BCP/Cu	1.15	23.33	83.3	22.37	≈80%, 120 h, RT ^d,g^	
**2FMPA-BT-CA**	ITO/2FMPA-BT-CA/(FA_0.17_MA_0.94_PbI_3.11_)_0.95_(PbCl_2_)_0.05_/C_60_/BCP/Cu	1.14	22.81	83.1	21.68	>74%, 120 h, RT ^d,g^	
**Cz-CA**	ITO/Cz-CA/Cs_0.05_(FA_0.92_MA_0.08_)_0.95_Pb(I_0.92_Br_0.08_)_3_/PCBM/C_60_/Ag	~1.06	~23.2	~83.0	~20.0	-	[85]
**Cz-Ph-CA**	ITO/MPA-Ph-CA/Cs_0.05_(FA_0.92_MA_0.08_)_0.95_Pb(I_0.92_Br_0.08_)_3_/PCBM/C_60_/Ag	~1.11	~23.0	~83.0	~20.2	-	
**TPA-CA**	ITO/TPA-CA/Cs_0.05_(FA_0.92_MA_0.08_)_0.95_Pb(I_0.92_Br_0.08_)_3_/PCBM/C_60_/Ag	~1.06	~22.4	~81.0	~17.5	-	
**TPA-Ph-CA**	ITO/TPA-Ph-CA/Cs_0.05_(FA_0.92_MA_0.08_)_0.95_Pb(I_0.92_Br_0.08_)_3_/PCBM/C_60_/Ag	~1.12	~23.0	~82.5	~20.0	--	
**MPA-CA**	ITO/MPA-CA/Cs_0.05_(FA_0.92_MA_0.08_)_0.95_Pb(I_0.92_Br_0.08_)_3_ /PCBM/C_60_/Ag	~1.10	~23.5	~81.0	~20.5	-	
**MPA-Ph-CA**	ITO/MPA-Ph-CA/Cs_0.05_(FA_0.92_MA_0.08_)_0.95_Pb(I_0.92_Br_0.08_)_3_/PCBM/C_60_/Ag	1.139	23.55	84.02	22.53	95%, 800 h, 45 °C ^c,f^	
**PQxD**	ITO/PQxD/FASnI_3_/C_60_/BCP/Ag	0.542	19.28	68.1	7.1	≈90%,1600 h ^b,f^	[86]
**TQxD**	ITO/TQxD/FASnI_3_/C_60_/BCP/Ag	0.574	21.05	68.8	8.3	≈90%,1600 h ^b,f^	

^a)^ maintained performance of its initial PCE after a specific time; ^b)^ dark condition; ^c)^ simulated 1 sun AM 1.5G illumination; ^d)^ relative humidity 50%; ^e)^ relative humidity 72%; ^f)^ inert gas; ^g)^ ambient air.

### 2.5. Other SAMs

**Figure 5:**
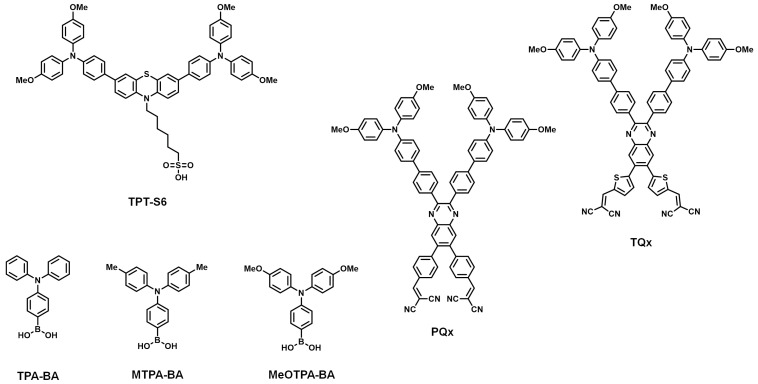
Molecular structures of other anchoring group-based SAMs.

Besides the PA, CA, and CAA anchoring groups, several other anchoring groups have been introduced into SAMs (Figure 5). In the Section 2.2, an SO_3_H anchoring group was introduced into SAM to compare its binding effect [66]. Although **TPT-S6**, with a SO_3_H anchoring group, showed lower device stability and decreased PCE due to its acidity, it demonstrated potential by yielding a PCE value of 16.16%, about 4% higher than that without any anchoring group, which was 11.97%. In the “Section 2.4.”, Afraj et al. synthesized X-shaped SAMs (**PQx** and **TQx**) with a quinoxaline-based head with a dicyano anchoring group [86]. The **PQx** and **TQx** contain a benzene and thiophene linker group. Although **TQxD,** with a CAA anchoring group, exhibited the highest PCE value of 8.3%, **TQx** also showed a promising PCE value of 8.0% by demonstrating the best hole mobility among the synthesized SAMs [87].

In contrast to previous studies which primarily focused on the PA, CA, and CAA anchoring groups, there have been some reports introducing entirely new anchoring groups using innovative strategies. Guo et al. introduced a boric acid anchoring group into SAMs [88]. Importantly, the low acidity of a boric acid anchor significantly reduced corrosion at the ITO electrode. They developed **MTPA-BA**, **MeOTPA-BA**, and **TPA-BA**, each with different alkyl groups on the triphenylamine head group. Among them, **MTPA-BA**-based devices achieved an impressive PCE close to 23%, with a high FF of 85.2%. Notably, **MTPA-BA**-based PSCs achieved a T_90_ of over 2400 h, demonstrating a 5-fold increase in shelf-life compared to the control SAM-based HTM (**2PACz**). This demonstrates the great potential of boric acid as the anchoring group of SAMs for PSCs. The photovoltaic properties of PSCs in this section are summarized in Table 4.

## 3. Conclusions and Outlook

**Figure 6:**
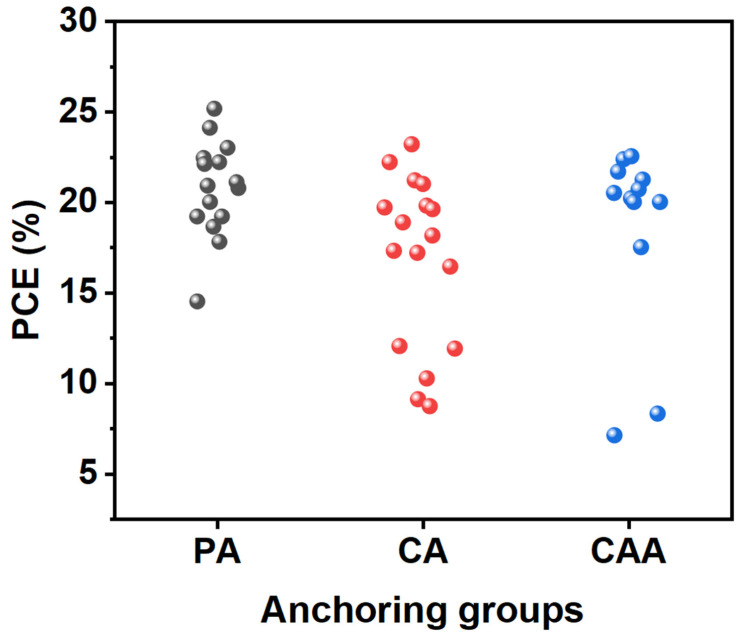
Statistical distribution of PCE values of PSCs based on SAMs depending on the PA, CA, and CAA anchoring groups.

SAM-based HTMs have significantly increased PCEs in inverted PSCs. These SAMs, forming an ultrathin and uniform coating on the TCO substrate, offer notable advantages on the modification of WF at the TCO surface, straightforward large-area processing, and the reduction in surface resistance and process costs. In this review, we predominantly emphasized the synthesis of SAMs according to their anchoring groups and summarized their *V*_oc_, *J*_sc_, FF, and PCEs with respect to device stability. Figure 6 shows the statistical distribution of PCE values of SAM-based PSCs depending on their PA, CA, and CAA anchoring groups.

However, the chemical structure of the reported SAMs is limited to a few anchoring groups, mostly categorized in PA, CA, and CAA groups. Thus, the development of new anchoring groups and the diversification of SAM structures seem to be truly necessary for SAM-based HTMs. Currently, the bonding strength and acidity of the anchoring groups are not carefully considered when building SAMs, but those seems to be important in determining the stability of the devices and the hole-transporting properties at the interfaces of the TCO and perovskite layers. In addition, most head groups are based on benzene ring-based moieties such as carbazole, triphenylamine, and phenothiazine derivatives. Other heterocyclic rings such as furan, thiophene, and selenophene also need to be explored.

Furthermore, recently, solvent-free vacuum deposition stood out for its advanced and consistent coating capabilities. The vacuum deposition of 2PACz HTM enhanced the PCEs of PSCs compared to those of conventional solution-processed devices [89]. Currently, this vacuum deposition method is not widely utilized, and, thus, the synthesis of vacuum-deposition SAM-based HTMs provides a great opportunity for the development of high-performance PSCs.

In summary, we provided a brief overview of the structure, features, and synthesis of SAM-based HTMs according to their anchoring groups. We hope that this review provides some insight into the different types of head groups and anchoring groups and consequently hope to contribute to further progress in the development of SAM-based PSCs.

## Figures and Tables

**Table 1:** Photovoltaic properties of PSCs using **PA**-based SAMs.

HTM	Device Structure	V_OC_ [V]	J_SC_ [mA/cm^−2^]	FF [%]	PCE [%]	Device Stability ^a^	Refs.
**V1036**	ITO/V1036/C4/Cs_0.05_(MA_0.17_FA_0.83_)_0.95_Pb(I_0.83_Br_0.17_)_3_/C_60_/BCP/Cu	1.09	21.9	81.0	17.8	~94%, 180 d, RT ^b,f^	[60]
**MeO-2PACz**	ITO/MeO–2PACz/Cs_0.05_(MA_0.17_FA_0.83_)_0.95_Pb(I_0.83_Br_0.17_)_3_/C_60_/BCP/Cu	1.144	22.2	79.3	19.2	>97%, 11 h, ~40 °C ^c,f^	[61]
**2PACz**	ITO/2PACz/C4/Cs_0.05_(MA_0.17_FA_0.83_)_0.95_Pb(I_0.83_Br_0.17_)_3_/C_60_/BCP/Cu	1.188	21.9	80.2	20.9	~97%, 11 h, ~40 °C ^c,f^	
**Me-4PACz**	ITO/Me-4PACz/Cs_0.05_(FA_0.77_MA_0.23_)_0.95_Pb(I_0.77_Br_0.23_)_3_/C_60_/SnO_2_/Ag	1.15	20.3	84	20.0	95.5%, 300 h, RT ^c,d,g^	[62]
**Br-2EPT**	ITO/Br–2EPT/Cs_0.05_(FA_0.92_MA_0.08_)_0.95_Pb(I_0.92_Br_0.08_)_3_/C_60_/BCP/Cu	1.09	25.11	82.0	22.44	107.4%, 100 h, RT ^c,e,g^	[63]
**BCBBr-C4PA**	ITO/BCBBr-C4PA/FA_0.8_Cs_0.2_Pb(I_0.6_Br_0.4_)_3_/C_60_/ALD-SnO_2_/Cu	1.286	17.54	82.61	18.63	>90%, 250 h ^c,f^	[64]
**CbzPh**	ITO/CbzPh/Cs_0.05_MA_0.15_FA_0.80_PbI_3_/C_60_/BCP/Ag	1.17	23.43	73.06	19.2	91%, 120 h ^c,f^	[65]
**CbzNaph**	ITO/CbzNaph/Cs_0.05_MA_0.15_FA_0.80_PbI_3_/C_60_/BCP/Ag	1.17	24.69	83.39	24.1	97%, 120 h ^c,f^	
**4PACz**	ITO/4PACz/Cs_0.05_MA_0.15_FA_0.80_PbI_3_/C_60_/BCP/Ag	1.07	23.20	58.43	14.5	88%, 120 h ^c,f^	
**TPT-P6**	ITO/TPT-P6/Cs_0.05_MA_0.12_FA_0.83_Pb(I_0.85_Br_0.15_)_3_/C_60_/BCP/Ag	1.125	23.20	80.38	20.77	>90%, 200 h ^c,d,g^	[66]
**MPA-CPA**	ITO/MPA-CPA/Cs_0.05_(FA_0.95_MA_0.05_)_0.95_Pb(I_0.95_Br_0.05_)_3_/C_60_/BCP/Ag	1.20	24.8	84.5	25.16	>90%, 2000 h, ~45 °C ^c,d,g^	[67]
**3PATAT-C3**	ITO/3PATAT-C3/Cs_0.05_FA_0.80_MA_0.15_PbI_2.75_Br_0.25_/EDAI2/C_60_/BCP/Ag	1.13	24.5	83	23.0	~100%, 2000 h, RT ^b,f^	[68]
**2PATAT-C3**	ITO/2PATAT-C3/Cs_0.05_FA_0.80_MA_0.15_PbI_2.75_Br_0.25_/EDAI2/C_60_/BCP/Ag	1.14	23.3	83	22.2	-	
**1PATAT-C3**	ITO/1PATAT-C3/Cs_0.05_FA_0.80_MA_0.15_PbI_2.75_Br_0.25_/EDAI2/C_60_/BCP/Ag	1.06	24.0	82	21.1	-	
**3PATAT-C4**	ITO/3PATAT-C4/Cs_0.05_FA_0.80_MA_0.15_PbI_2.75_Br_0.25_/EDAI2/C_60_/BCP/Ag	1.14	23.3	83	22.1	-	

^a)^ maintained performance of its initial PCE after a specific time; ^b)^ dark condition; ^c)^ simulated 1 sun AM 1.5G illumination; ^d)^ relative humidity 30–40%; ^e)^ relative humidity 15–25%; ^f)^ inert gas; ^g)^ ambient air.

**Table 2:** Photovoltaic properties of PSCs using **CA**-based SAMs.

HTM	Device Structure	V_OC_ [V]	J_SC_ [mA/cm^−2^]	FF [%]	PCE [%]	Device Stability ^a^	Refs.
**MC-43**	ITO/MC-43/Cs_0.05_(MA_0.17_FA_0.83_)_0.95_Pb(I_0.83_Br_0.17_)_3_/PCBM/Ag	1.07	20.3	80.0	17.3	90%, 20 d ^b^	[74]
**EA-41**	ITO/EA-41/MAPbI_3_/PCBM/Ca/Ag	1.019	17.33	69.86	11.89	-	[71]
**EA-46**	ITO/EA-46/MAPbI_3_/PCBM/Ca/Ag	1.006	15.70	62.18	10.24	-	
**EA-49**	ITO/EA-49/MAPbI_3_/PCBM/Ca/Ag	1.024	17.95	68.15	12.03	-	
**EA-50**	ITO/EA-50/MAPbI_3_/PCBM/Ca/Ag	1.035	16.91	56.30	9.09	-	
**EA-56**	ITO/EA-56/MAPbI_3_/PCBM/Ca/Ag	1.023	15.70	56.56	8.71	-	
**TPA-PT-C6**	ITO/co-assembled TPA-PT-C-6/MAPbI_3_/PCBM/BCP/Ag	1.04	21.8	77.4	17.19	89%, 120 d ^c,e,g^	[75]
**EADR03**	ITO/EADR03/Cs_0.05_FA_0.79_MA_0.16_Pb(I_0.84_Br_0.16_)_3_ /LiF/C_60_/BCP/NaF/Cu	1.156	22.9	80.0	21.2	~95%, 250 h, RT ^c,f^	[76]
**EADR04**	TO/EADR04/Cs_0.05_FA_0.79_MA_0.16_Pb(I_0.84_Br_0.16_)_3_/ LiF/C_60_/BCP/NaF/Cu	1.164	22.6	80.0	21.0	~91%, 250 h, RT ^c,f^	
**RC-24**	ITO/RC-24/CsFAMA/C_60_/BCP/Cu	1.12	22.3	79	19.8	~97%, 120 s	[77]
**RC-25**	ITO/RC-25/CsFAMA/C_60_/BCP/Cu	1.12	22.1	79	19.6	~96%, 120 s	
**RC-.34**	ITO/RC-34/CsFAMA/C_60_/BCP/Cu	1.11	22.5	79	19.7	~95%, 120 s	
**TPT-C6**	ITO/TPT-C6/Cs_0.05_MA_0.12_FA_0.83_Pb(I_0.85_Br_0.15_)_3_/ C_60_/BCP/Ag	1.042	23.14	74.16	18.87		[66]
**AC-1**	ITO/AC-1/CsFAMAPb/PCBM/BCP/Ag	1.084	18.83	80.49	16.43	85%, 30 d, RT ^b,d,g^	[79]
**AC-3**	ITO/AC-3/CsFAMAPb/PCBM/BCP/Ag	1.108	24.27	82.56	22.20	90%, 30 d, RT ^b,d,g^	
**AC-5**	ITO/AC-5/CsFAMAPb/PCBM/BCP/Ag	1.130	24.42	84.05	23.19	91%, 30 d, RT ^b,d,g^	
**Spiro-Acid**	ITO/Spiro-Acid/Cs_0.05_(FA_0.85_MA_0.15_)_0.95_Pb(I_0.85_Br_0.15_)_3_	0.990	22.20	82.6	18.15	≈100%,100 h ^c,f^	[80]

^a)^ maintained performance of its initial PCE after a specific time; ^b)^ dark condition; ^c)^ simulated 1 sun AM 1.5G illumination; ^d)^ relative humidity 20–30%; ^e)^ relative humidity 30–40%; ^f)^ inert gas; ^g)^ ambient air.

**Table 4:** Photovoltaic properties of PSCs using other SAMs.

HTM	Device Structure	V_OC_ [V]	J_SC_ [mA/cm^−2^]	FF [%]	PCE [%]	Device Stability ^a^	Refs.
**TPT-S6**	ITO/TPT-S6/Cs_0.05_MA_0.12_FA_0.83_Pb(I_0.85_Br_0.15_)_3_/C_60_/BCP/Ag	0.943	21.15	73.33	16.16	50.5~%, 80 d ^d,g^	[66]
**PQx**	ITO/PQx/FASnI_3_/C_60_/BCP/Ag	0.455	19.97	66.6	6.1	≈60%, 1600 h ^b,f^	[86]
**TQx**	ITO/TQx/FASnI_3_/C_60_/BCP/Ag	0.546	21.30	69.0	8.0	≈90%, 1600 h ^b,f^	
**MTPA-BA**	ITO/MTPA-BA/Cs_0.05_(FA_0.95_MA_0.05_)_0.95_Pb(I_0.95_Br_0.05_)_3_/PCBM/C_60_/BCP/Ag	1.14	23.24	85.2	22.62	90%, 20 h 40 °C ^c,e,g^	[88]

^a)^ maintained performance of its initial PCE after a specific time; ^b)^ dark condition; ^c)^ simulated 1 sun AM 1.5G illumination; ^d)^ relative humidity 30%; ^e)^ relative humidity 40%; ^f)^ inert gas; ^g)^ ambient air.

## Data Availability

Data are available upon request.
